# Gender differences in the associations between childhood adversity and psychopathology in the general population

**DOI:** 10.1007/s00127-023-02546-5

**Published:** 2023-08-25

**Authors:** Thanavadee Prachason, Irem Mutlu, Laura Fusar-Poli, Claudia Menne-Lothmann, Jeroen Decoster, Ruud van Winkel, Dina Collip, Philippe Delespaul, Marc De Hert, Catherine Derom, Evert Thiery, Nele Jacobs, Marieke Wichers, Jim van Os, Bart P. F. Rutten, Lotta-Katrin Pries, Sinan Guloksuz

**Affiliations:** 1 Department of Psychiatry, Faculty of Medicine Ramathibodi Hospital, Mahidol University, Bangkok, Thailand; 2 Department of Psychiatry and Neuropsychology, School for Mental Health and Neuroscience, Maastricht University Medical Center, P.O. Box 616, 6200 MD Maastricht, The Netherlands; 3 Institute of Graduate Programs, Department of Clinical Psychology, Istanbul Bilgi University, Istanbul, Turkey; 4 Department of Brain and Behavioral Sciences, University of Pavia, Pavia, Italy; 5 Psychiatric Care Sint-Kamillus, Bierbeek, Belgium; 6 Department of Neurosciences, University Psychiatric Centre KU Leuven, KU Leuven, Leuven, Belgium; 7 University Psychiatric Centre Katholieke Universiteit Leuven, Kortenberg, Belgium; 8 Department of Neurosciences, Centre for Clinical Psychiatry, Katholieke Universiteit Leuven, Leuven, Belgium; 9 Leuven Brain Institute, Katholieke Universiteit Leuven, Leuven, Belgium; 10 Antwerp Health Law and Ethics Chair, University of Antwerp, Antwerp, Belgium; 11 Department of Obstetrics and Gynecology, Ghent University Hospitals, Ghent University, Ghent, Belgium; 12 Department of Neurology, Ghent University Hospital, Ghent University, Ghent, Belgium; 13 Faculty of Psychology, Open University of the Netherlands, Heerlen, The Netherlands; 14 Department of Psychiatry, Interdisciplinary Center Psychopathology and Emotion Regulation (ICPE), University of Groningen, University Medical Center Groningen, Groningen, The Netherlands; 15 Department of Psychiatry, Brain Centre Rudolf Magnus, University Medical Centre Utrecht, Utrecht, The Netherlands; 16 Department of Psychosis Studies, Institute of Psychiatry, King’s Health Partners, King’s College London, London, UK; 17 Department of Psychiatry, Yale School of Medicine, New Haven, CT USA

**Keywords:** Childhood adversity, Abuse, Neglect, Gender differences, Psychopathology, General population

## Abstract

**Purpose:**

To explore gender differences of the associations between childhood adversity (CA) subtypes and psychiatric symptoms in the general population.

**Methods:**

Data of 791 participants were retrieved from a general population twin cohort. The Symptom Checklist-90 Revised (SCL-90) and the Childhood Trauma Questionnaire were used to assess overall psychopathology with nine symptom domains scores and total CA with exposure to five CA subtypes, respectively. The associations between CA and psychopathology were analyzed in men and women separately and were subsequently compared.

**Results:**

Total CA was associated with total SCL-90 and all symptom domains without significant gender differences. However, the analyses of CA subtypes showed that the association between emotional abuse and total SCL-90 was stronger in women compared to men [*χ*^2^(1) = 4.10, *P* = 0.043]. Sexual abuse was significantly associated with total SCL-90 in women, but emotional neglect and physical neglect were associated with total SCL-90 in men. Exploratory analyses of CA subtypes and SCL-90 subdomains confirmed the pattern of gender-specific associations. In women, emotional abuse was associated with all symptom domains, and sexual abuse was associated with all except phobic anxiety and interpersonal sensitivity. In men, emotional neglect was associated with depression, and physical neglect was associated with phobic anxiety, anxiety, interpersonal sensitivity, obsessive–compulsive, paranoid ideation, and hostility subdomains.

**Conclusion:**

CA is a trans-syndromal risk factor regardless of gender. However, differential associations between CA subtypes and symptom manifestation might exist. Abuse might be particularly associated with psychopathology in women, whereas neglect might be associated with psychopathology in men.

**Supplementary Information:**

The online version contains supplementary material available at 10.1007/s00127-023-02546-5.

## Introduction

Childhood adversity (CA) is a major risk factor for multiple health problems worldwide. The prevalence of CA is around one-eighth to one-third in non-clinical populations across the globe [1] and even higher among people with mental health problems [2, 3]. Studies consistently revealed that adverse experiences during childhood are linked to all mental disorders, as well as a lifetime admixture of psychopathology in clinical and subclinical populations [4–6]. The population attributable fraction of CA exposure is estimated to be around 33% for psychosis and 59% for depression as well as anxiety [7, 8]. Moreover, a history of CA is a poor prognostic factor shared among major psychiatric disorders [9–12], emphasizing its significant impact on mental health outcomes.

According to the World Health Organization, CA is defined as abuse and neglect experienced during childhood or adolescence, including all types of physical and emotional ill-treatment, sexual abuse, neglect, as well as all forms of exploitation that result in actual or potential harm to a child’s health, survival, development, and dignity within their social and family context [13]. Although the link between CA exposure and poor physical and mental health has been consistently reported across all types of adverse experiences [14, 15], accumulating evidence suggests that different types of CA exposure could lead to distinctive pathways to mental health problems. For example, children exposed to violence, but not deprivation, show worse adaptation to emotional conflict and tend to assume ambiguous cues as hostile [16, 17], suggesting their impaired cognitive ability related to social information processing. Indeed, poor social-cognitive performance, such as difficulties in understanding others’ thoughts and intentions, and low predicting accuracy of others’ emotions, is not only associated with exposure to interpersonal violence but also mediates its relationship with externalizing behaviors in adolescents [18]. On the contrary, the deprivation of socioemotional and cognitive input was consistently found to be associated with poorer language skills [19–21] and impaired executive functions in children [22]. Interestingly, these deficits were shown to specifically link childhood deprivation, but not abuse, to ADHD symptoms and general psychopathology [23–25]. Altogether, these pieces of evidence suggest that subtype differentiation is necessary to unravel the differential contributions of CA subtypes to different psychopathology.

Adding more complexity to the features of CA, accumulating evidence demonstrates that women and men are neither equally exposed nor similarly susceptible to different CA subtypes [1, 26]. A meta-analysis of studies in non-clinical samples found that, on average, women reported a history of childhood sexual abuse twice as often as men [1]. Furthermore, men and women show different clinical outcomes and biological consequences in response to CA. A meta-analysis of studies in China found that female participants exposed to physical abuse are more prone to show externalizing behaviors, whereas male participants exposed to emotional abuse are more likely to have internalizing problems [27]. A large community youth cohort study showed that women exposed to a high number of traumatic stressful events show more anxiety and phobia symptoms compared to men [28]. On the contrary, men are more likely to manifest psychosis spectrum symptoms and externalizing behaviors when being exposed to assaults [28]. Neurobiological studies also suggest gender-dependent associations between CA and gray-matter volume (GMV) of certain brain regions. For instance, Dragan et al. (2019) found negative associations between the number of self-reported CA events and GMV in the left inferior parietal lobe and the right precentral gyrus specifically in women [29]. These areas are involved in negative emotional processing, emotion regulation, and self-evaluation. On the other hand, a negative association between the degree of CA exposure and GMV in the right fusiform gyrus, involved in face processing, was found in men [29]. Altogether, these findings suggest that CA exposure might be linked to symptom manifestation in men and women differently in a subtype-specific manner.

Nevertheless, studies on the influences of different CA subtypes in men and women are relatively limited compared to studies on cumulative exposure to CA, overall abuse, and overall neglect. Furthermore, co-exposure to multiple forms of adversity, which is a common phenomenon [5, 30–32], is normally not taken into account. In this study, we performed a systematic analysis of five CA subtypes (i.e., physical abuse, emotional abuse, sexual abuse, physical neglect, and emotional neglect) to investigate their associations with different psychopathology domains while accounting for their co-exposure. The associations were investigated separately in men and women and were compared to determine gender differences of the associations.

## Methods

The dataset used in this study was derived from the TwinssCan Project. A detailed description of enrollment and data collection was previously described elsewhere [33]. Briefly, participants were recruited from the East Flanders Prospective Twin Survey [34], a prospective population-based, multi-birth registry situated in Flanders, Belgium. Those fulfilling the inclusion criteria were invited to participate in the TwinssCan project [35], a longitudinal study collecting data of twins aged 15–35 years as well as their siblings and parents. The first assessment was performed from April 2010 to April 2014 [36]. All participants gave written informed consent. For participants below the age of 18, parent(s) also signed informed consent. Participants were excluded if they had a pervasive mental disorder as indicated by caregivers. The local ethics committee (Commissie Medische Ethiek van de Universitaire ziekenhuizen KU Leuven, Nr. B32220107766) approved the study. Data of 821 twins and siblings collected at the first wave of the TwinssCan project were included in the present study. Thirty participants were excluded from our analysis due to missing information regarding psychopathology or CA exposures (see Table S1), leaving 791 participants for the analyses.

### Measurements

#### Symptoms

The Symptom Checklist-90 Revised (SCL-90) [37], a 90-item self-report questionnaire, was used to assess overall psychopathology and nine symptom domains: psychoticism, paranoid ideation, anxiety, depression, somatization, obsessive–compulsive, interpersonal sensitivity, hostility, and phobic anxiety. Respondents were asked to rate the extent to which they were bothered by each symptom in the past week based on a 5-point Likert scale ranging from ‘not at all’ to ‘very much’. The SCL-90 Global Severity Index (hereafter: total SCL-90), ranging from 0 to 4, was derived by averaging the scores of all SCL-90 items [37]. The nine symptom domain scores were similarly derived by averaging all items per symptom domain [37]. Cronbach’s alpha coefficients of the SCL-90 scores in the original study ranged from 0.77 to 0.90 [37].

#### Childhood adversity

CA was assessed using the Childhood Trauma Questionnaire (CTQ) [38], which consists of 28 items rated on a 5-point Likert scale to assess five CA subtypes: physical abuse, emotional abuse, sexual abuse, physical neglect, and emotional neglect. All items were summed to reflect overall CA exposure (Hereafter: total CA). Cronbach’s alpha coefficients of CA subtypes in the original study ranged from 0.81 to 0.86 for physical abuse, 0.84 to 0.89 for emotional abuse, 0.92 to 0.95 for sexual abuse, 0.61 to 0.78 for physical neglect, and 0.85 to 0.91 for emotional neglect [38]. The manual of the CTQ suggests three severity cut-off scores (low, moderate, and severe) for each subscale [39, 40]. Consistent with previous work [41–43], the lowest cut-off scores were used to determine binary exposure to the CA subtypes: ≥ 9 for emotional abuse, ≥ 8 for physical abuse, ≥ 6 for sexual abuse, ≥ 10 for emotional neglect, and ≥ 8 for physical neglect.

### Statistical analyses

Data analyses were performed using Stata version 13.0 [44]. To test the gender-specific association between CA and psychopathology, we applied gender-stratified linear regression analyses with CA exposures as independent variables and psychopathology scores as dependent variables. For our primary analyses, we first tested the associations between total CA and total SCL-90 as well as the nine symptom domains in men and women separately. Following this, we tested the associations between all five CA subtypes and total SCL-90 in a mutually adjusted model accounting for the co-occurrence of other CA subtypes. As exploratory analyses, the associations of the five CA subtypes on each SCL-90 subdomain scores (i.e., psychoticism, paranoid ideation, anxiety, depression, somatization, obsessive–compulsive, interpersonal sensitivity, hostility, and phobic anxiety) were similarly tested. Statistical significance was set at *P* < 0.05 for the analyses using total SCL-90 as the outcome and Bonferroni-corrected* P* < 0.006 for the exploratory analyses using SCL-90 subdomains as the outcomes. In all models, to account for intrafamily correlation, standard errors (*SE*) were corrected for clustering of siblings within the same family using the Stata “cluster” option. Total SCL-90 and the symptom dimension scores were transformed using a square-root function and all analyses were adjusted for age. To capture the relative contribution of each explaining variable in the model, the Stata “Shapley2” post-estimation command [45] was used to calculate the Shorrocks–Shapley decomposition of *R*^2^ [46]. The Chow’s test [47] was used to compare the regression coefficients of the analyses between men and women.

As sensitivity analyses, we repeated the main analyses while bootstrapping the data to ensure that the associations were not inflated by the family structure of this sample. More specifically, 2000 resampled datasets were generated by randomly selecting one participant from each family strata while allowing for replacement. Bootstrap results are reported in the supplements.

## Results

Table S1 shows the number of missing reports for the included variables for men and women separately. After listwise deletion, data from 274 monozygotic twins, 474 dizygotic twins, and 43 siblings from 384 families were used in this analysis. About 60% of the participants were female and the age range of the dataset was 15–34 years. Table 1 summarizes the included variables for men and women separately.

**Table 1:** The characteristics of the participants

	Men (*n* = 314)	Women (*n* = 477)
Age (y), *M* (SD)	16.9 (2.8)	17.8 (4.0)
Zygosity, *n* (%)		
Monozygotic twins	97^a^ (30.9)	177^b^ (37.1)
Dizygotic twins	204^c^ (65.0)	270^d^ (56.6)
Sibling	13 (4.1)	30 (6.3)
Total SCL-90, *M* (SD)	0.43 (0.40)	0.50 (0.44)
Phobic anxiety	0.16 (0.37)	0.22 (0.44)
Anxiety	0.36 (0.45)	0.45 (0.54)
Depression	0.45 (0.51)	0.58 (0.58)
Interpersonal sensitivity	0.53 (0.51)	0.67 (0.58)
Somatization	0.44 (0.45)	0.53 (0.51)
Obsessive–compulsive	0.67 (0.55)	0.69 (0.59)
Paranoid ideation	0.44 (0.54)	0.46 (0.58)
Hostility	0.42 (0.46)	0.42 (0.49)
Psychoticism	0.26 (0.40)	0.26 (0.37)
Total CA, *M* (SD)	34.7 (8.2)	33.8 (8.8)
CA subtypes, *n* (%)		
Emotional abuse	102 (32.5)	145 (30.4)
Physical abuse	21 (6.7)	14 (2.9)
Sexual abuse	18 (5.7)	36 (7.6)
Emotional neglect	163 (51.9)	176 (36.9)
Physical neglect	55 (17.5)	74 (15.5)
Number of CA subtype exposure, *n* (%)		
None	111 (35.4)	225 (47.2)
One	104 (33.1)	124 (26.0)
Two	59 (18.8)	80 (16.8)
Three or more	40 (12.7)	48 (10.1)

^a^from 50 twin-pairs

^b^from 91 twin-pairs

^c^from 47 same-sex twin-pairs and 107 opposite-sex twin-pairs

^d^from 77 same-sex twin-pairs and 107 opposite-sex twin-pairs

*SCL-90* Symptom Checklist-90 Revised, *CA* childhood adversity, *M* mean, *SD* standard deviation

### Gender-stratified association between total childhood adversity and psychopathology

Total CA explained 15.4% and 12.9% of the variance of total SCL-90 in men (*B* = 0.013, *SE* = 0.003, *P* < 0.001) and women (*B* = 0.011, *SE* = 0.002, *P* < 0.001), respectively (Table 2). The sensitivity analysis confirmed the results (Table S2). The follow-up analysis revealed no significant gender difference of the association between total CA and total SCL-90 (*χ*^2^ = 0.23, df = 1, *P* = 0.630, Table 2).

**Table 2:** Gender-stratified associations between the total childhood adversity and psychopathology

Outcome	Men				Women				Gender difference in *B*	
	*B*	*SE*	*P* value	% Variance explained by total CA	*B*	*SE*	*P* value	% Variance explained by total CA	*χ*^2^, df(1)	*P* value
Total SCL-90	0.013	0.003	** < 0.001**	15.4	0.011	0.002	** < 0.001**	12.9	0.23	0.630
Phobic anxiety	0.016	0.003	** < 0.001**	15.7	0.010	0.003	** < 0.001**	5.3	2.24	0.134
Anxiety	0.014	0.003	** < 0.001**	10.4	0.013	0.002	** < 0.001**	8.3	0.19	0.664
Depression	0.016	0.003	** < 0.001**	12.7	0.012	0.002	** < 0.001**	8.5	0.92	0.339
Interpersonal sensitivity	0.014	0.003	** < 0.001**	10.2	0.012	0.002	** < 0.001**	7.2	0.41	0.522
Somatization	0.012	0.003	** < 0.001**	7.7	0.012	0.002	** < 0.001**	8.6	0.001	0.982
Obsessive–compulsive	0.011	0.003	**0.001**	5.8	0.012	0.002	** < 0.001**	7.8	0.05	0.823
Paranoid ideation	0.016	0.003	** < 0.001**	10.1	0.017	0.002	** < 0.001**	11.7	0.04	0.841
Hostility	0.015	0.003	** < 0.001**	10.8	0.011	0.002	** < 0.001**	8.2	0.83	0.362
Psychoticism	0.017	0.003	** < 0.001**	15.7	0.014	0.002	** < 0.001**	13.7	0.37	0.541

Age was added as a covariate in all models. Statistical significance (Bonferroni-corrected *P* < 0.006) is presented in bold. *SCL-90* Symptom Checklist-90 Revised, *CA* childhood adversity, *B* unstandardized regression coefficient, *SE* clustered standard error

**Fig. 1:**
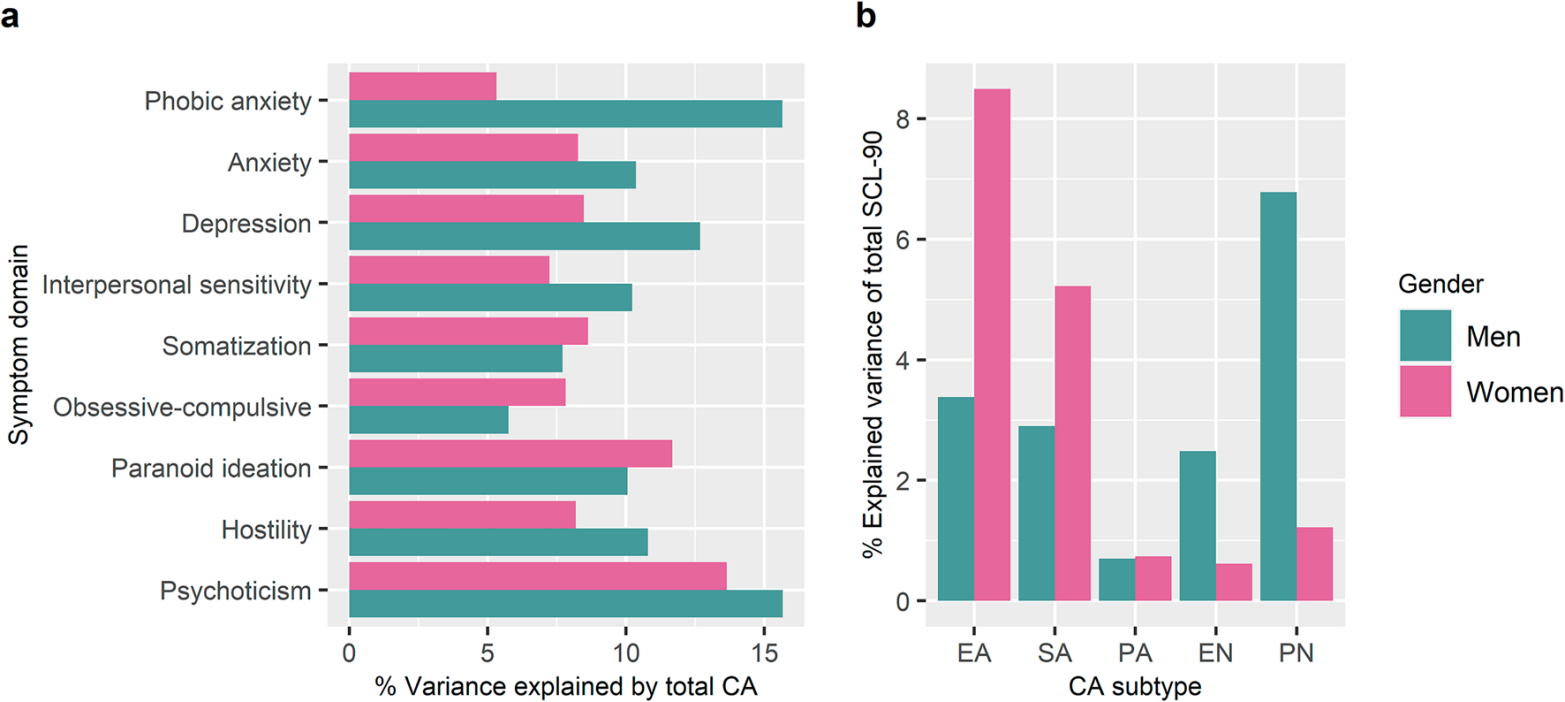
Gender-stratified associations between childhood adversity and psychopathology: **a** % variance of each symptom domain explained by total CA; **b** % variance of total SCL-90 explained by exposure to CA subtypes.; *EA* emotional abuse, *PA* physical abuse, *SA* sexual abuse, *EN* emotional neglect, *PN* physical neglect

The sequential analyses of the psychopathology subdomains revealed that total CA was significantly associated with all symptom domains in men and women (all Bonferroni-corrected *P* < 0.006, Table 2). However, the order of hits from the largest to lowest explained variance differed for men and women (Fig. 1). In men, the variances explained by total CA (Shapley value) ranged from 5.8% to 15.7%. It explained the largest variances for psychoticism (15.7%) and phobic anxiety (15.7%), followed by depression (12.7%), hostility (10.8%), anxiety (10.4%), interpersonal sensitivity (10.2%), paranoid ideation (10.1%), somatization (7.7%), and obsessive–compulsive domains (5.8%). In women, the explained variance ranged from 5.3% to 13.7%. Total CA explained the largest variances for psychoticism (13.7%), followed by paranoid ideation (11.7%), somatization (8.6%), depression (8.5%), anxiety (8.3%), hostility (8.2%), obsessive–compulsive (7.8%), interpersonal sensitivity (7.2%), and phobic anxiety domains (5.3%). The sensitivity analyses confirmed the results (Table S2). The follow-up comparison between men and women indicated no statistically significant differences of the associations between total CA and any of the symptom domains (Table 2).

### Gender-stratified association between childhood adversity subtypes and psychopathology

An analysis of the association between CA subtypes and general psychopathology showed gender-specific patterns. In men, physical neglect (*B* = 0.167, *SE* = 0.043, *P* < 0.001), emotional neglect (*B* = 0.061, *SE* = 0.027, *P* = 0.026), and emotional abuse (*B* = 0.080, *SE* = 0.035, *P* = 0.023) were significantly associated with total SCL-90. In women, sexual abuse (*B* = 0.217, *SE* = 0.053, *P* < 0.001) and emotional abuse (*B* = 0.173, *SE* = 0.030, *P* < 0.001) were significantly associated with total SCL-90 (Table 3). No significant associations between other CA subtypes and total SCL-90 were found. The sensitivity analyses confirmed the significant findings (Table S3). The follow-up analyses revealed that the association between emotional abuse and total SCL-90 was significantly more prominent among women than men (*χ*^2^ = 4.10, df = 1, *P* = 0.043). No significant gender differences were found for any of the other CA subtypes (Table 3).

**Table 3:** Gender-stratified associations between the five subtypes of childhood adversity and the total psychopathology

Explaining variables	Men				Women				Gender difference in *B*	
	*B*	*SE*	*P* value	% Variance explained	*B*	*SE*	*P* value	% Variance explained	*χ*^2^, df(1)	*P* value
Emotional abuse	0.080	0.035	**0.023**	3.4	0.173	0.030	** < 0.001**	8.5	4.10	**0.043**
Physical abuse	–0.004	0.080	0.964	0.7	0.053	0.097	0.587	0.7	0.20	0.653
Sexual abuse	0.153	0.091	0.094	2.9	0.217	0.053	** < 0.001**	5.2	0.36	0.549
Emotional neglect	0.061	0.027	**0.026**	2.5	–0.017	0.029	0.566	0.6	3.81	0.051
Physical neglect	0.167	0.043	** < 0.001**	6.8	0.059	0.039	0.127	1.2	3.36	0.067

Age was added as a covariate in the model. Statistical significance (*P* < 0.05) is presented in bold. *B* unstandardized regression coefficient, *SE* clustered standard error

**Fig. 2:**
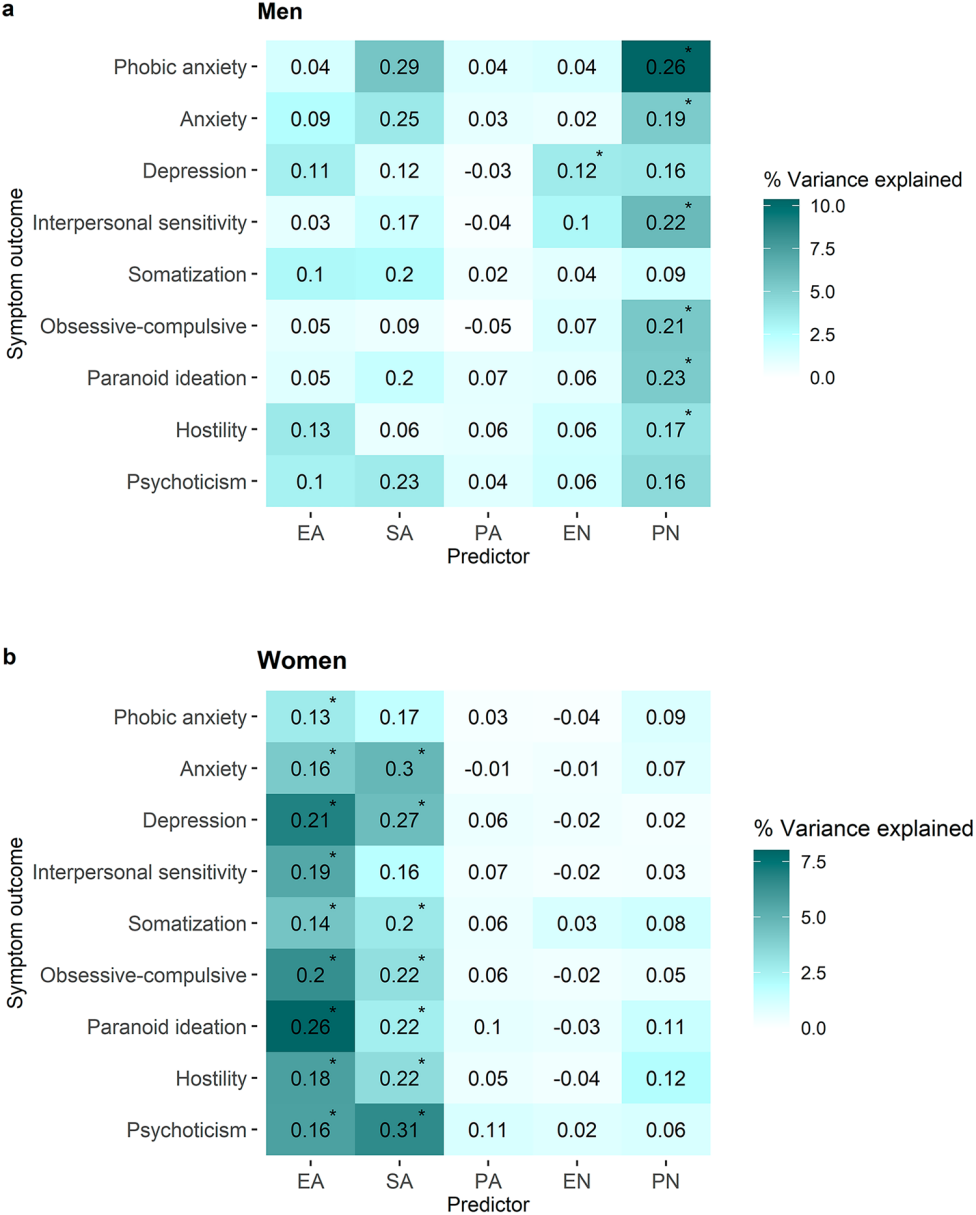
Gender-stratified associations between the five subtypes of childhood adversity and symptom domains in **a** men and **b** women. Age was added as a covariate in all models. The unstandardized regression coefficients (*B*) of each CA subtype were shown as numbers. The significant associations (Bonferroni-corrected *P* < 0.006) were marked by asterisks. The percentages of variance of symptom domains explained by each CA subtype were illustrated by heatmap. *EA* emotional abuse, *PA* physical abuse, *SA* sexual abuse, *EN* emotional neglect, *PN* physical neglect

Finally, the exploratory analyses testing the association between CA subtypes and psychopathology subdomains (Table S4) confirmed the gender-specific patterns of associations. For visualization, Fig. 2 demonstrates the pattern of association showing the explained variances for men and women separately. In men, physical neglect was significantly associated with six symptom domains (i.e., phobic anxiety, anxiety, interpersonal sensitivity, obsessive–compulsive, paranoid ideation, and hostility) and emotional neglect was significantly associated with depression (Bonferroni-corrected *P* < 0.006). No other statistically significant associations between CA subtypes and symptom domains were found (Fig. 2 and Table S4). In women, emotional abuse was associated with all symptom domains and sexual abuse was associated with seven symptom domains (i.e., anxiety, depression, somatization, obsessive–compulsive, paranoid ideation, hostility, and psychoticism). No other statistically significant associations between CA subtypes and symptom domains were found (Fig. 2 and Table S4). The sensitivity analyses converged with the findings (Table S5). The follow-up analyses revealed that the association between emotional abuse and paranoid ideation was significantly stronger in women than men (*χ*^2^ = 8.5, df = 1, *P* = 0.004; Table S4). No other significant gender differences were observed (Table S4).

## Discussion

Previous studies have suggested that CA exposure might be differently linked to psychopathology in men and women in a subtype-specific manner [24, 27, 28]. However, simultaneous analyses of multiple CA subtypes to account for their co-occurrence are scarce despite its commonplace. In this study, we explored gender-specific associations of overall CA and concurrent exposure to five CA subtypes (i.e., physical abuse, emotional abuse, sexual abuse, physical neglect, and emotional neglect) on population-level psychopathology (i.e., psychoticism, paranoid ideation, anxiety, depression, somatization, obsessive–compulsive, interpersonal sensitivity, hostility, and phobic anxiety). We found that total CA was positively associated with general psychopathology as well as all symptom domains in both genders. Nevertheless, simultaneous analyses of the five CA subtypes revealed gender-specific patterns of associations. Controlling for age and concurrent exposure to other CA subtypes, emotional neglect and physical neglect were specifically associated with general psychopathology in men. On the other hand, emotional abuse showed a significantly stronger association with general psychopathology in women. Sexual abuse was also significantly associated with general psychopathology specifically in women. Analyses of the psychopathology domains confirmed these gender-specific differences. The associations with emotional abuse and sexual abuse were statistically significant across almost all symptom domains in women but none in men. Conversely, physical neglect was significantly associated with six symptom domains in men but none in women. Besides, emotional neglect showed a significant association with depression only in men but not women.

In agreement with previous studies [28, 48, 49], our findings confirmed the non-specific influence of early life adversity across all symptom domains in men and women. Nonetheless, several observations should be noted. First, among all symptom domains, CA exposure showed the strongest association with psychoticism symptoms with the largest explained variance in both genders. Second, although no significant gender differences for any of the associations were found, the overall order of hits from the gender-stratified analyses differed for men and women. For instance, the association between CA and phobic anxiety was among the top hits with the largest explained variance in men, whereas in women, phobic anxiety was linked to the lowest explained variance compared to the other symptom domains. This is in contrast to a recent study by Barzilay and colleagues (2019) showing that women exposed to a high number of traumatic stressful events have more anxiety and phobia symptoms compared to men [28]. However, the traumatic events in this study included natural disaster, accidents, direct or indirect exposure to physical assault, and sexual abuse, which may be more similar to abuse rather than neglect domains of CA. Concordantly, our exploratory analyses of CA subtypes revealed that phobic anxiety was specifically associated with emotional abuse in women and with physical neglect in men. Such differential pattern of associations of CA subtypes in men and women could explain the absence of gender differences in the analyses with total CA.

Overall, the explorative analyses with the CA subtypes showed different association patterns for men and women. The associations between abuse subtypes (particularly sexual and emotional abuse) and psychopathology were generally stronger and more extensive in women compared to men. This pattern was consistent across all analyses. However, as an exception, physical abuse was not significantly associated with any psychopathology subdomain in either gender, which might be due to the low prevalence of this adversity subtype in our dataset. Notwithstanding, the finding that abuse subtypes were more relevant for women generally aligns with the previous studies related to anxiety and depressive symptoms. A large community survey in Canada found that the associations between childhood abuse (i.e., physical and sexual abuse) and lifetime psychiatric disorders, including anxiety and depressive disorders, are stronger in women than men [50]. This trend was supported by a meta-analysis of population-based studies, showing that the influences of abuse subtypes (i.e., physical and sexual abuse) on depressive and anxiety symptoms tended to be larger for women compared to men [51]. On the contrary, another recent meta-analysis of studies in population-representative samples found that all forms of CA are associated with an increased risk of depressive and anxiety disorders with no gender differences [14]. Despite inconclusive findings in epidemiological studies, a neuroimaging study in medically healthy young adults showed that childhood abuse, but not neglect, predicts adult hippocampal volume in female participants [52]. As alterations of hippocampal volume are found in a variety of psychiatric disorders [53], this result supports the idea that women might be particularly susceptible to the development of psychopathology after exposure to childhood abuse.

Apart from depression and anxiety, several studies indicated that abuse subtypes may be important for the pathoetiology of psychosis expression particularly in women. In two samples of clinical psychosis, childhood abuse was found to be associated with earlier age of psychosis onset particularly among women [54, 55]. Another study in patients with schizophrenia and schizoaffective disorder showed that women exposed to childhood physical abuse have more positive and depressive symptoms compared to men with or without trauma exposure [56]. Moreover, a path analysis in female participants with subthreshold psychotic symptoms revealed that high exposure to childhood threats is associated with increased stress perception, which subsequently predicted salivary morning cortisol [57]. This association pattern was not found in male participants. This is relevant as enhanced threat perception has been proposed as a mechanism involving social information processing that links abuse subtypes of maltreatment to the development of transdiagnostic psychopathology [58, 59]. Although some inconsistent reports exist [26], accumulating evidence supports the idea that childhood abuse plays an important role in the pathoetiology of mental health problems particularly in women.

Regarding childhood neglect, we also observed differential patterns of associations between genders. Overall, childhood neglect seemed to be more relevant for the pathoetiology of mental health in men. In other words, physical neglect and emotional neglect were positively associated with total SCL-90 in men, whereas neither neglect domains were associated with the overall psychopathology in women. As the prevalence of physical neglect in men and women was comparable in our dataset and the prevalence of emotional neglect was high for both genders, such distinctive association patterns are unlikely to be underlain by the gender difference in prevalence of trauma exposure. Furthermore, the findings converged with the follow-up analyses on psychopathology subdomains, showing significant associations of physical and emotional neglect with certain symptom domains among men, but null associations in women. Although some previous studies do not show such gender-specific pattern [31, 49], several studies support the idea that neglect domains are more relevant for the pathoetiology of mental health in men. A longitudinal study in Danish offspring of mothers of individuals with schizophrenia showed that institutionalization, which is linked to parental absence, increased symptoms of thought disorder specifically among men [60]. Furthermore, neuroimaging studies revealed that the volume of the hippocampus and postcentral gyrus gray matter are negatively associated with childhood neglect or deprivation experience only among male participants [52, 61]. Finally, translational evidence is provided by a recent animal study [62]. The study showed that maternal separation, which is simulated by an experimental model simulating stress associated with loss of parental care, results in an anxiety-like outcome and changes in stress physiology only among male mice. In summary, combined with previous evidence, our findings indicate that men may be more susceptible to neglect and depriving experiences than women.

### Strengths and limitations

We used a systematic approach to provide detailed insight into gender-specific patterns of associations between CA subtypes and psychopathology in the general population. Nevertheless, several limitations should be noted.

First, the dataset was derived from a twin cohort recruited from the province of East Flanders in Belgium with little variation in ethnicity. Therefore, generalizability to other general populations might be limited. Replication studies in other ethnically diverse general population cohorts are required to confirm the differential association patterns observed in this study.

Second, we used a retrospective self-report questionnaire to assess CA exposure, which can be subjected to recall and reporting biases [63]. However, it was suggested that the bias of retrospective reports of CA is more likely under-reporting rather than over-reporting [64, 65], possibly leading to false-negative rather than false-positive findings. Furthermore, studies in large population-representative cohorts have shown that retrospective and prospective reports of CA exposure are both associated with psychiatric problems in late adolescence as well as various health and social outcomes assessed subjectively and objectively in adulthood [66, 67]. Retrospective self-reported CA appears to be more strongly associated with psychiatric problems than prospective informant-reported CA regardless of the method of mental health assessment [66–68]. Such evidence corroborates the clinical meaningfulness of the current analyses. Nonetheless, as other sources of information related to CA exposure are unavailable in this dataset, the comparability of our findings and those based on a prospective assessment of CA exposure need to be elucidated in future studies.

Third, as we adjusted for other CA subtypes, the results highlighted the CA subtypes that might have significant weight when mutually controlling for other CA subtypes. This is particularly important as epidemiological studies have demonstrated that co-exposure to various forms of maltreatment is common [5, 30–32] and that exposures are correlated [69]. Furthermore, around one-third of the participants in our dataset experienced more than one type of CA. Nonetheless, this does not mean that the other CA subtypes cannot have an impact. Further studies are needed to investigate the gender-specific effects of CA subtypes when occurring in isolation and in combination. Moreover, as other potential risk and confounding factors, including socio-economic status, physical activity, medication/substance use, and significant life events, were not adjusted for in the current analysis, future studies that include these factors in the models as covariates or apply exposomic approach to investigate aggregated influences of multiple exposures acting in concert are warranted [70].

Fourth, the current sample included twins as well as their siblings. Siblings growing up in the same household will likely have been exposed to similar childhood experiences as well as other vulnerability factors (e.g., genetics and other environmental exposures) that are commonly linked to psychopathology. Thus, associations in this study might have been inflated by the family structure in our dataset. However, we estimated the standard errors adjusting for family structure, which is a commonly used approach in twin studies [71, 72]. In addition to that, we conducted sensitivity analyses by bootstrapping the dataset with unrelated individuals. The results from the sensitivity analyses converged with the main findings.

Fifth, although stratified analyses indicated several differences in the associations between CA subtypes and psychopathology in men and women, the subsequent comparison between groups was not always significant. These inconsistencies might indicate that there might be Type I errors for the stratified analyses or Type II errors for the comparison analyses. However, given the consistency of the overall pattern of association in the stratified analyses and our strict Bonferroni-correction approach, it is unlikely that Type I errors occurred. Thus, the fact that we found fewer significant associations in the comparison analyses might indicate that the sample was too small for this subsequent analysis involving multiple testing. Similarly, the analyses with physical abuse might have been underpowered in our study given that the prevalence of physical abuse was relatively low (*n* = 21 in men, *n* = 14 in women). Nonetheless, we reported the results of these explorative analyses for transparency and to encourage further studies to replicate the results in pre-registered hypothesis testing research using larger samples.

Finally, our analyses included cross-sectional data, precluding an assumption of a causal relationship between CA and psychopathology. Furthermore, the onset and duration of CA exposure, which are moderating factors of the impact of trauma exposure [73–75], were not specified in this study. Therefore, longitudinal approaches that also capture the onset and course of CA exposure as well as genetically informed causal inference methods are required to elaborate further on the potentially causal gender-specific links between CA and psychopathology.

## Conclusion

In conclusion, we confirmed trans-syndromal associations of CA with multiple domains of psychopathology in both men and women. Additionally, we uncovered gender-specific patterns of susceptibility to abuse (especially sexual and emotional) for women and neglect (especially physical) for men. These findings highlight the need to take gender-specific patterns into account when evaluating the effects of CA subtypes on psychopathology.

### Supplementary Information

Below is the link to the electronic supplementary material.

Supplementary file1 (DOCX 50 KB)

## Data Availability

Data are available upon request from the first author and with proper approval from appropriate institutional review boards.
