# Supplementary material for: Gender differences in the associations between childhood adversity and psychopathology in the general population
Source: Soc Psychiatry Psychiatr Epidemiol. 2023 Aug 25;59(5):847–58. doi: 10.1007/s00127-023-02546-5 (PMC11087312; doi:10.1007/s00127-023-02546-5)
Supplement: Supplementary file 1 — Supplementary file1 (DOCX 50 KB) [file 127_2023_2546_MOESM1_ESM.docx]

**Article Title:** Gender differences in the associations between childhood adversity and psychopathology in the general population

**Journal Name:** Social Psychiatry and Psychiatric Epidemiology

**Authors:** Thanavadee Prachason^a,b^; Irem Mutlu^c^; Laura Fusar-Poli^d^, Claudia Menne-Lothmann^b^; Jeroen Decoster^b,e^; Ruud van Winkel^b,f^; Dina Collip^b^; Philippe Delespaul^b^; Marc De Hert^g,h,i,j^; Catherine Derom^k^; Evert Thiery^l^; Nele Jacobs^b,m^; Marieke Wichers^b,n^; Jim van Os^b,o,p^; Bart P. F. Rutten^b^; Lotta-Katrin Pries^b*^; Sinan Guloksuz^b,q*^

**Affiliations:**

^a^ Department of Psychiatry, Faculty of Medicine Ramathibodi Hospital, Mahidol University, Bangkok, Thailand

^b^ Department of Psychiatry and Neuropsychology, School for Mental Health and Neuroscience, Maastricht University Medical Center, Maastricht, the Netherlands

^c^ Institute of Graduate Programs, Department of Clinical Psychology, Istanbul Bilgi University, Istanbul, Turkey

^d^ Department of Brain and Behavioral Sciences, University of Pavia, Pavia, Italy

^e^ Psychiatric Care Sint-Kamillus, Bierbeek, Belgium

^f^ Department of Neurosciences, University Psychiatric Centre KU Leuven, KU Leuven, Belgium

^g^ University Psychiatric Centre Katholieke Universiteit Leuven, Kortenberg, Belgium

^h^ Department of Neurosciences, Centre for Clinical Psychiatry, Katholieke Universiteit Leuven, Belgium

^i^ Leuven Brain Institute, Katholieke Universiteit Leuven, Leuven, Belgium

^j^ Antwerp Health Law and Ethics Chair, University of Antwerp, Antwerp, Belgium

^k^ Department of Obstetrics and Gynecology, Ghent University Hospitals, Ghent University, Ghent, Belgium

^l^ Department of Neurology, Ghent University Hospital, Ghent University, Ghent, Belgium

^m^ Faculty of Psychology, Open University of the Netherlands, Heerlen, the Netherlands

^n^ Department of Psychiatry, Interdisciplinary Center Psychopathology and Emotion Regulation (ICPE), University of Groningen, University Medical Center Groningen, Groningen, the Netherlands

^o^ Department of Psychiatry, Brain Centre Rudolf Magnus, University Medical Centre Utrecht, Utrecht, the Netherlands

^p^ Department of Psychosis Studies, Institute of Psychiatry, King’s Health Partners, King’s College London, London, UK

^q^ Department of Psychiatry, Yale School of Medicine, New Haven, CT, USA

**Shared last author*

**Corresponding author’s e-mail:** [sinan.guloksuz@maastrichtuniversity.nl](mailto:sinan.guloksuz@maastrichtuniversity.nl)

**Table S1** The number of missing reports in men and women

| Variables | Number of missing reports | |
| --- | --- | --- |
|  | Men | Women |
| Age | 0 | 0 |
| Family number | 0 | 0 |
| Total SCL-90 | 17 | 13 |
| Phobic anxiety | 16 | 13 |
| Anxiety | 17 | 13 |
| Depression | 16 | 12 |
| Interpersonal sensitivity | 16 | 13 |
| Somatization | 16 | 13 |
| Obsessive- compulsive | 16 | 13 |
| Paranoid ideation | 17 | 13 |
| Hostility | 17 | 13 |
| Psychoticism | 17 | 13 |
| Total CA | 15 | 10 |
| Emotional abuse | 15 | 10 |
| Physical abuse | 14 | 10 |
| Sexual abuse | 15 | 10 |
| Emotional neglect | 15 | 10 |
| Physical neglect | 15 | 10 |

SCL-90 = Symptom Checklist-90 Revised; CA = childhood adversity

**Table S2** Gender-stratified associations between the total childhood adversity and psychopathology in the bootstrapped sample

| Outcome | Men | | | Women | | |
| --- | --- | --- | --- | --- | --- | --- |
|  | *B* | *SE* | *P*-value | *B* | *SE* | *P*-value |
| Total SCL-90 | 0.014 | 0.002 | **<.001** | 0.012 | 0.001 | **<.001** |
| Phobic anxiety | 0.016 | 0.002 | **<.001** | 0.012 | 0.001 | **<.001** |
| Anxiety | 0.014 | 0.002 | **<.001** | 0.013 | 0.001 | **<.001** |
| Depression | 0.017 | 0.002 | **<.001** | 0.013 | 0.001 | **<.001** |
| Interpersonal sensitivity | 0.015 | 0.002 | **<.001** | 0.012 | 0.001 | **<.001** |
| Somatization | 0.012 | 0.002 | **<.001** | 0.012 | 0.001 | **<.001** |
| Obsessive- compulsive | 0.011 | 0.002 | **<.001** | 0.012 | 0.001 | **<.001** |
| Paranoid ideation | 0.017 | 0.002 | **<.001** | 0.018 | 0.001 | **<.001** |
| Hostility | 0.016 | 0.002 | **<.001** | 0.013 | 0.001 | **<.001** |
| Psychoticism | 0.017 | 0.002 | **<.001** | 0.015 | 0.001 | **<.001** |

Note: Age was added as a covariate in all models. Statistical significance after Bonferroni’s correction (*P* < .006) is presented in bold. SCL-90 = Symptom Checklist-90 Revised; *B* = unstandardized regression coefficient; *SE* = clustered standard error

**Table S3** Gender-stratified associations between the five subtypes of childhood adversity and the total psychopathology in the bootstrapped sample

| Explaining variables | Men | | | Women | | |
| --- | --- | --- | --- | --- | --- | --- |
|  | *B* | *SE* | *P*-value | *B* | *SE* | *P*-value |
| Emotional abuse | 0.096 | 0.024 | **<.001** | 0.171 | 0.020 | **<.001** |
| Physical abuse | 0.041 | 0.044 | .934 | 0.088 | 0.043 | .216 |
| Sexual abuse | 0.184 | 0.060 | **.010** | 0.195 | 0.040 | **<.001** |
| Emotional neglect | 0.055 | 0.019 | **.002** | -0.021 | 0.019 | .376 |
| Physical neglect | 0.142 | 0.028 | **<.001** | 0.068 | 0.028 | **.033** |

Note: Age was added as a covariate in the model. Statistical significance (*P* < .05) is presented in bold. *B* = unstandardized regression coefficient; *SE* = clustered standard error

**Table S4** Gender-stratified associations between the five subtypes of childhood trauma and psychopathology subdomains in the original sample

| Outcome | Explaining variables | Men | | | | Women | | | | Gender difference in *B* | |
| --- | --- | --- | --- | --- | --- | --- | --- | --- | --- | --- | --- |
|  |  | *B* | *SE* | *P*-value | % Variance explained | *B* | *SE* | *P*-value | % Variance explained | ꭓ^2^, df(1) | *P*-value |
| Phobic anxiety | EA | 0.037 | 0.041 | .366 | 1.53 | 0.128 | 0.043 | **.003** | 2.79 | 2.52 | .112 |
|  | PA | 0.037 | 0.089 | .674 | 1.23 | 0.035 | 0.139 | .804 | 0.21 | 0.000 | .986 |
|  | SA | 0.288 | 0.123 | .020 | 5.57 | 0.165 | 0.083 | .048 | 1.69 | 0.64 | .423 |
|  | EN | 0.040 | 0.032 | .212 | 1.36 | -0.040 | 0.035 | .253 | 0.17 | 3.06 | .080 |
|  | PN | 0.265 | 0.058 | **<.001** | 10.4 | 0.090 | 0.051 | .082 | 1.02 | 5.43 | .020 |
| Anxiety | EA | 0.092 | 0.050 | .065 | 2.59 | 0.156 | 0.042 | **<.001** | 4.09 | 1.06 | .303 |
|  | PA | 0.029 | 0.096 | .765 | 0.91 | -0.013 | 0.110 | .906 | 0.23 | 0.08 | .772 |
|  | SA | 0.248 | 0.106 | .020 | 3.69 | 0.302 | 0.080 | **<.001** | 4.85 | 0.15 | .699 |
|  | EN | 0.024 | 0.038 | .528 | 0.73 | -0.012 | 0.039 | .763 | 0.35 | 0.44 | .506 |
|  | PN | 0.195 | 0.060 | **.001** | 5.26 | 0.074 | 0.047 | .116 | 0.86 | 2.43 | .119 |
| Depression | EA | 0.114 | 0.047 | .016 | 3.32 | 0.212 | 0.039 | **<.001** | 6.95 | 2.63 | .105 |
|  | PA | -0.026 | 0.099 | .795 | 0.38 | 0.055 | 0.132 | .674 | 0.52 | 0.24 | .625 |
|  | SA | 0.123 | 0.118 | .301 | 1.31 | 0.274 | 0.069 | **<.001** | 4.60 | 1.15 | .283 |
|  | EN | 0.116 | 0.037 | **.002** | 3.62 | -0.021 | 0.039 | .589 | 0.39 | 6.56 | .011 |
|  | PN | 0.161 | 0.059 | .007 | 3.77 | 0.016 | 0.054 | .765 | 0.28 | 3.10 | .078 |
| Interpersonal sensitivity | EA | 0.035 | 0.047 | .460 | 0.96 | 0.194 | 0.044 | **<.001** | 5.47 | 6.44 | .011 |
|  | PA | -0.043 | 0.104 | .676 | 0.25 | 0.071 | 0.124 | .568 | 0.42 | 0.53 | .469 |
|  | SA | 0.169 | 0.099 | .090 | 1.82 | 0.164 | 0.065 | .012 | 1.85 | 0.001 | .969 |
|  | EN | 0.103 | 0.040 | .011 | 2.90 | -0.022 | 0.041 | .583 | 0.31 | 4.97 | .026 |
|  | PN | 0.218 | 0.054 | **<.001** | 6.12 | 0.031 | 0.055 | .572 | 0.35 | 5.84 | .016 |
| Somatization | EA | 0.102 | 0.044 | .022 | 2.85 | 0.138 | 0.039 | **<.001** | 4.33 | 0.40 | .528 |
|  | PA | 0.017 | 0.083 | .838 | 0.59 | 0.061 | 0.103 | .556 | 0.52 | 0.11 | .739 |
|  | SA | 0.201 | 0.094 | .034 | 2.70 | 0.199 | 0.061 | **.001** | 2.88 | 0.00 | .987 |
|  | EN | 0.043 | 0.039 | .267 | 1.09 | 0.031 | 0.037 | .393 | 1.09 | 0.05 | .825 |
|  | PN | 0.089 | 0.055 | .105 | 1.69 | 0.078 | 0.043 | .070 | 1.34 | 0.02 | .877 |
| Obsessive-compulsive | EA | 0.049 | 0.049 | .321 | 0.93 | 0.196 | 0.041 | **<.001** | 6.41 | 5.41 | .020 |
|  | PA | -0.051 | 0.088 | .564 | 0.14 | 0.060 | 0.109 | .581 | 0.49 | 0.64 | .425 |
|  | SA | 0.090 | 0.096 | .349 | 0.69 | 0.218 | 0.068 | **.001** | 3.25 | 1.15 | .283 |
|  | EN | 0.065 | 0.039 | .095 | 1.35 | -0.020 | 0.040 | .622 | 0.41 | 2.36 | .124 |
|  | PN | 0.214 | 0.052 | **<.001** | 5.39 | 0.045 | 0.050 | .367 | 0.59 | 5.50 | .019 |
| Paranoid ideation | EA | 0.055 | 0.054 | .314 | 1.18 | 0.264 | 0.048 | **<.001** | 8.02 | 8.50 | **.004** |
|  | PA | 0.072 | 0.102 | .480 | 0.95 | 0.100 | 0.118 | .395 | 0.68 | 0.03 | .853 |
|  | SA | 0.196 | 0.118 | .099 | 2.03 | 0.225 | 0.062 | **<.001** | 2.63 | 0.05 | .829 |
|  | EN | 0.056 | 0.045 | .222 | 1.03 | -0.027 | 0.046 | .562 | 0.59 | 1.65 | .200 |
|  | PN | 0.233 | 0.063 | **<.001** | 5.22 | 0.111 | 0.058 | .056 | 1.50 | 2.01 | .156 |
| Hostility | EA | 0.127 | 0.049 | .010 | 3.69 | 0.177 | 0.038 | **<.001** | 5.76 | 0.64 | .423 |
|  | PA | 0.062 | 0.094 | .511 | 0.91 | 0.051 | 0.133 | .701 | 0.53 | 0.004 | .948 |
|  | SA | 0.059 | 0.088 | .502 | 0.82 | 0.217 | 0.061 | **<.001** | 3.39 | 2.14 | .144 |
|  | EN | 0.063 | 0.042 | .134 | 1.64 | -0.044 | 0.034 | .203 | 0.36 | 3.95 | .047 |
|  | PN | 0.170 | 0.051 | **.001** | 4.00 | 0.119 | 0.051 | .019 | 2.00 | 0.49 | .485 |
| Psychoticism | EA | 0.098 | 0.045 | .030 | 3.18 | 0.158 | 0.039 | **<.001** | 5.73 | 1.07 | .302 |
|  | PA | 0.043 | 0.103 | .677 | 1.12 | 0.110 | 0.115 | .338 | 1.11 | 0.18 | .668 |
|  | SA | 0.225 | 0.112 | .045 | 3.60 | 0.306 | 0.062 | **<.001** | 6.58 | 0.40 | .527 |
|  | EN | 0.059 | 0.036 | .103 | 1.75 | 0.016 | 0.035 | .640 | 0.93 | 0.75 | .386 |
|  | PN | 0.163 | 0.062 | .009 | 4.47 | 0.060 | 0.044 | .172 | 1.10 | 1.79 | .181 |

Note: Age was added as a covariate in all models. Statistical significance (Bonferroni-corrected *P* < .006) is presented in bold. *B* = unstandardized regression coefficient; *SE* = clustered standard error; EA = emotional abuse; PA = physical abuse; SA = sexual abuse; EN = emotional neglect; PN = physical neglect

**Table S5** Gender-stratified associations between the five subtypes of childhood adversity and psychopathology subdomains in the bootstrapped sample

| Outcome | Explaining variables | Men | | | Women | | |
| --- | --- | --- | --- | --- | --- | --- | --- |
|  |  | *B* | *SE* | *P*-value | *B* | *SE* | *P*-value |
| Phobic anxiety | EA | 0.027 | 0.027 | .156 | 0.133 | 0.031 | **<.001** |
|  | PA | 0.081 | 0.048 | .438 | 0.194 | 0.072 | .632 |
|  | SA | 0.372 | 0.100 | **.004** | 0.177 | 0.072 | .022 |
|  | EN | 0.030 | 0.022 | .063 | -0.048 | 0.027 | .132 |
|  | PN | 0.234 | 0.039 | **<.001** | 0.096 | 0.035 | .010 |
| Anxiety | EA | 0.100 | 0.029 | **.002** | 0.170 | 0.031 | **<.001** |
|  | PA | 0.082 | 0.049 | .563 | 0.040 | 0.050 | 0.797 |
|  | SA | 0.278 | 0.076 | **.001** | 0.251 | 0.065 | **<.001** |
|  | EN | 0.028 | 0.025 | .338 | -0.015 | 0.027 | .659 |
|  | PN | 0.149 | 0.035 | **<.001** | 0.065 | 0.037 | .047 |
| Depression | EA | 0.127 | 0.031 | **<.001** | 0.214 | 0.027 | **<.001** |
|  | PA | 0.016 | 0.053 | .631 | 0.082 | 0.067 | .406 |
|  | SA | 0.168 | 0.074 | .096 | 0.241 | 0.054 | **<.001** |
|  | EN | 0.117 | 0.026 | **<.001** | -0.029 | 0.026 | .417 |
|  | PN | 0.126 | 0.040 | **<.001** | 0.028 | 0.039 | .675 |
| Interpersonal sensitivity | EA | 0.037 | 0.028 | .220 | 0.188 | 0.027 | **<.001** |
|  | PA | 0.032 | 0.053 | .415 | 0.073 | 0.064 | .272 |
|  | SA | 0.187 | 0.066 | .010 | 0.141 | 0.048 | **<.001** |
|  | EN | 0.099 | 0.024 | **<.001** | -0.038 | 0.028 | .426 |
|  | PN | 0.195 | 0.029 | **<.001** | 0.051 | 0.038 | .409 |
| Somatization | EA | 0.108 | 0.025 | **<.001** | 0.143 | 0.023 | **<.001** |
|  | PA | 0.043 | 0.047 | .719 | 0.078 | 0.054 | .255 |
|  | SA | 0.263 | 0.057 | **<.001** | 0.170 | 0.050 | **<.001** |
|  | EN | 0.034 | 0.022 | .055 | 0.033 | 0.024 | .186 |
|  | PN | 0.071 | 0.034 | .010 | 0.096 | 0.030 | .009 |
| Obsessive-compulsive | EA | 0.077 | 0.032 | .129 | 0.193 | 0.027 | **<.001** |
|  | PA | 0.004 | 0.056 | .363 | 0.079 | 0.061 | .329 |
|  | SA | 0.104 | 0.059 | .125 | 0.175 | 0.052 | **<.001** |
|  | EN | 0.056 | 0.027 | .015 | -0.023 | 0.024 | .421 |
|  | PN | 0.186 | 0.031 | **<.001** | 0.054 | 0.034 | .190 |
| Paranoid ideation | EA | 0.068 | 0.039 | .159 | 0.254 | 0.032 | **<.001** |
|  | PA | 0.140 | 0.062 | .243 | 0.119 | 0.062 | .106 |
|  | SA | 0.194 | 0.079 | .013 | 0.221 | 0.048 | **<.001** |
|  | EN | 0.051 | 0.029 | .059 | -0.026 | 0.031 | .379 |
|  | PN | 0.216 | 0.039 | **<.001** | 0.132 | 0.042 | .008 |
| Hostility | EA | 0.139 | 0.027 | **<.001** | 0.188 | 0.026 | **<.001** |
|  | PA | 0.110 | 0.050 | .210 | 0.071 | 0.051 | .319 |
|  | SA | 0.103 | 0.079 | .455 | 0.220 | 0.041 | **<.001** |
|  | EN | 0.063 | 0.025 | .012 | -0.051 | 0.025 | .084 |
|  | PN | 0.160 | 0.036 | **<.001** | 0.119 | 0.033 | **<.001** |
| Psychoticism | EA | 0.115 | 0.028 | **<.001** | 0.145 | 0.025 | **<.001** |
|  | PA | 0.113 | 0.049 | .377 | 0.153 | 0.061 | .070 |
|  | SA | 0.237 | 0.073 | **.002** | 0.309 | 0.049 | **<.001** |
|  | EN | 0.051 | 0.022 | **.007** | 0.005 | 0.025 | .512 |
|  | PN | 0.135 | 0.033 | **<.001** | 0.083 | 0.036 | .098 |

Note: Age was added as a covariate in all models. Statistical significance (Bonferroni-corrected *P* < .006) is presented in bold. *B* = unstandardized regression coefficient; *SE* = clustered standard error; EA = emotional abuse; PA = physical abuse; SA = sexual abuse; EN = emotional neglect; PN = physical neglect
